# Bioactivity of essential oils extracted from *Cupressus macrocarpa* branchlets and *Corymbia citriodora* leaves grown in Egypt

**DOI:** 10.1186/s12906-018-2085-0

**Published:** 2018-01-22

**Authors:** Mohamed Z. M. Salem, Hosam O. Elansary, Hayssam M. Ali, Ahmed A. El-Settawy, Mohamed S. Elshikh, Eslam M. Abdel-Salam, Krystyna Skalicka-Woźniak

**Affiliations:** 1 Forestry and Wood Technology Department, Faculty of Agriculture (EL-Shatby), Alexandria University, Aflaton St., El-Shatby, P.O. Box 21545, Alexandria, Egypt; 2 Department of Floriculture, Ornamental Horticulture and Garden Design, Faculty of Agriculture (El-Shatby), Alexandria University, Alexandria, Egypt; 3 Department of Geography, Environmental Management and Energy Studies, University of Johannesburg, APK campus, 2006, Johannesburg, South Africa; 4 Botany and Microbiology Department, College of Science, King Saud University, P.O. Box 2455, Riyadh, 11451 Saudi Arabia; 5 Timber Trees Research Department, Sabahia Horticulture Research Station, Horticulture Research Institute, Agriculture Research Center, Alexandria, Egypt; 6 Department of Pharmacognosy with Medicinal Plant Unit, Medical University of Lublin, Lublin, Poland

**Keywords:** Antibacterial activity, Antifungal activity, Antioxidant activity, *Corymbia citriodora*, *Cupressus macrocarpa*, Essential oil

## Abstract

**Background:**

*Cupressus macrocarpa* Hartw and *Corymbia citriodora* (Hook.) K.D. Hill & L.A.S. Johnson, widely grown in many subtropical areas, are used for commercial purposes, such as in perfumery, cosmetics, and room fresheners. Their potential as a source of antimicrobial compounds may be useful in different applications.

**Methods:**

The chemical composition of essential oils (EOs) from *C. macrocarpa* branchlets and *C. citriodora* leaves was analyzed by using gas chromatography–mass spectrometry (GC/MS). Antibacterial and antifungal activities were assessed by the micro-dilution method to determine the minimum inhibitory concentrations (MICs), and minimum fungicidal concentrations (MFCs), and minimum bactericidal concentrations (MBCs). Further, the antioxidant capacity of the EOs was determined via 2,2′-diphenypicrylhydrazyl (DPPH) and *β*-carotene-linoleic acid assays.

**Results:**

Terpinen-4-ol (23.7%), α-phellandrene (19.2%), *α*-citronellol (17.3%), and citronellal were the major constituents of EO from *C. macrocarpa* branchlets, and *α*-citronellal (56%), *α*-citronellol (14.7%), citronellol acetate (12.3%), isopulegol, and eucalyptol were the primary constituents of EO from *C. citriodora* leaves. Antibacterial activity with MIC values of EO from *C. citriodora* leaves was ranged from 0.06 mg/mL to 0.20 mg/mL, and MBC from 0.12 mg/mL against *E. coli* to 0.41 mg/mL. EO from *C. macrocarpa* branchlets showed less activity against bacterial strains. The MIC values against tested fungi of the EO from *C. citriodora* ranged from 0.11 to 0.52 mg/mL while for EO from *C. macrocarpa* from 0.29 to 3.21 mg/mL. The MIC and MFC values of EOs against *P. funiculosum* were lower than those obtained from Ketoconazole (KTZ) (0.20; 0.45; 0.29 and 0.53 mg/mL, respectively, vs 0.21 and 0.41 mg/mL. Antioxidant activity of the EO from *C. citriodora* was higher than that of the positive control but lower than that of the standard butylhydroxytoluene (BHT) (IC_50_ = 5.1 ± 0.1 μg/mL).

**Conclusion:**

The results indicate that the EO from Egyptian trees such as *C. citriodora* leaves may possesses strong bactericidal and fungicidal activities and can be used as an agrochemical for controlling plant pathogens and in human disease management which will add crop additive value.

## Background

Essential oils (EOs) and their constituents have potential applications for use in food products as they have been shown to have antifungal, antibacterial, and antioxidant properties [1–6]. The side effects associated with synthetic antimicrobial and antioxidant products urged a global search for natural products, such as natural EOs, with multiuse options. EOs are moderate to strong antioxidants and preservatives used in food processing. They are also used as antimicrobial agents in food supplement production and the pharmaceutical industry [2, 7, 8].

The “Cypress” plants belong to the family Cupressaceae and are grown in many subtropical areas for commercial purposes, such as ornamentation, and as a source of wood-building material [9, 10]. *Cupressus macrocarpa* is an evergreen tree up to 23-m tall with horizontal branches [11]. Leaf EO from this plant is used against rheumatism, whooping cough, and styptic problems [12]. Several authors [11, 13–17] have described the EOs of *C. macrocarpa*. Zavarin et al. [18] focused on monoterpenes found in oil needles, while Cool [16], focused on the sesquiterpene compounds. A larger amount of monoterpenes, as compared to sesquiterpenes or diterpenes, was detected in the EOs of the branchlets of *C. macrocarpa* [18]. The major compounds identified in volatile oil from the cone of *C. macrocarpa* Hartwig from Nilgiris, India were terpinel-4-ol, dinopol, *α*-pinene, and *β*-pinene [11]. Recently Fahed et al. [19] reported that the EOs of *C. macrocarpa* has strong activity against specific dermal fungi.

*Eucalyptus citriodora* (Hook.) or *Corymbia citriodora* (Hook.) K.D.Hill & L.A.S. Johnson is widely used in perfumery, cosmetics, and room fresheners. For example, extracts of dried leaves resulted by hot water are traditionally used for many purposes like antipyretic remedies, anti-inflammatory, and analgesic as well as for the symptoms of respiratory infections, such as cold, and flu [20, 21].

The EOs of *C. citriodora* can be used as an antibacterial, antifungal, anticandidal, antioxidant, and antitrypanosomal, and also have insecticidal, acaricidal, herbicidal, analgesic, and anti-inflammatory activities [21–26]. Citronellal, *β*-citronellol, and isopulegol are monoterpenoids reported as major components in the leaf EO of *C. citriodora* growing in Chandigarh, India [26, 27]. However, 6-octenal was reported as the major constituent in the leaf oil of *C. citriodora* from Nigeria [25]; *α*-pinene, *β*-pinene, sabinene, and *α*-thujene were reported to be minor constituents [28]. 3-Hexen-1-ol, *cis*-geraniol, citronellol acetate, 5-hepten-1-ol, 2,6-dimethyl, and citronellal were the major components in the leaf EO of *C. citriodora* grown in Zoological Garden in Giza-Egypt [29]. The EO of *C. citriodora* (lemon-scented eucalyptus) showed a wide spectrum of antifungal activity as well as activity against various pathogenic bacteria and yeasts [22, 30–39] resulting in that the EOs of some plants, including *C. citriodora,* have significant insecticidal activity against *Sitophilus zeamais*, however, 50% of the efficacy was lost 8 days after treatment.

In the framework of our continuing research on the EO composition and biological activities of Egyptian medicinal plants, we aimed to evaluate the biological activity of the EOs of *Corymbia citriodora* leaves and *Cupressus macrocarpa* “Citriodora” branchlets. For the first time full analysis of essential oils from both plants collected in Egypt was done as well as full characteristic of their antibacterial, antifungal activities against set of Gram-plus, Gram-minus as fungus was done. Additionally, antioxidant potential was evaluated.

## Methods

### Plant material

Air-dried materials of *Corymbia citriodora* leaves, Myrtaceae (from a plantation located at Alexandria-Cairo desert road (Albostan area), Alexandria, Egypt) and *Cupressus macrocarpa* Hartw branchlets “Citriodora” Cupressaceae (from Faculty of Agriculture Garden, Alexandria, Egypt) were used in the present study during 2016. The plants were identified by Prof. Ahmed A. El-Settawy (Head of Forestry and Wood Technology Department) and given the voucher numbers Zidan00312 and Zidan313, respectively at the Faculty of Agriculture, Alexandria University. The plants were further morphologically approved by Dr. Hosam Elansary at the department of Floriculture, Ornamental horticulture and Garden Design.

### Extraction of essential oils

Samples of *C. citriodora* leaves and *C. macrocarpa* branchlets were cut into small pieces (100 g) and hydro-distillated for 3 h, in a Clevenger apparatus [40]. The oil was collected and the mass of fresh weight of sample was measured (3.15 and 4.70 mL/100 g fresh weight, from *C. citriodora* and *C. macrocarpa*, respectively). The oil was kept dry in sealed Eppendorf tubes and stored at 4 °C prior for chemical analysis.

### GC/FID and GC/MS analysis of the EO

GC Ultra/Mass spectrophotometer ISQ (Thermo Scientific), a trace instrument equipped with an FID and a DB-5 narrow bore column (length 10 m × 0.1 mm ID, 0.17-μm film thickness; Agilent, Palo Alto, CA, USA) was used. Following the same conditions as described by Salem et al. [41]. 

Identification of the constituents was performed using an MS library search [42, 43] as well as calcualting the Retention indices (RIs). Computer matching was performed with the Wiley 275.L and Wiley 7 n.L libraries. GC-MS analysis of each of triplicate samples was repeated three times.

### Antibacterial activities

Both Gram-positive and Gram-negative bacteria were used for analyses. The Gram-positive bacteria included *Bacillus cereus* (clinical isolate), *Listeria monocytogenes* (ATCC 19113), *Micrococcus flavus* (ATCC 10240) and *Staphylococcus aureus* (ATCC 6538). The Gram-negative bacteria included *Dickeya solani* (D s0432–1), *Escherichia coli* (ATCC 35210), *Pectobacterium atrosepticum* (ATCC 33260), *Pectobacterium carotovorum* subsp. *carotovorum* (ATCC 15713), and *Pseudomonas aeruginosa* (D s0432–1). The micro-dilution method [44] was used to determine the MIC and minimum bactericidal concentration (MBC). The concentration of the bacteria was adjusted to 1.0 × 10^5^ CFU/mL by using sterile saline, and then stored at 4 °C. The essential oils were added to 100 μL Triptic Soy broth (TSB) containing a bacteria inoculum (1.0 × 10^5^ CFU/well) in a microtiter plate, then the MICs and MBCs were determined. The microplates were incubated at 37 °C for 24 h in a rotary shaker.

A serial sub-cultivation of 2 μL was placed in microtiter plates containing 100 μL of TSB for each well and incubated for 24 h to determine the MIC and MBC. The optical density was measured using a microplate manager at 655 nm. Experiments were completed in triplicate. Dimethyl sulfoxide (DMSO, 5%) and streptomycin (1 mg/mL) were used as negative and positive controls, respectively.

### Antifungal activities

The activities of EOs against several fungi, including *Aspergillus flavus* (ATCC 9643), *A. ochraceus* (ATCC 12066), *A. niger* (ATCC 6275), *Candida albicans* (ATCC 12066), *Penicillium funiculosum* (ATCC 56755) and *P. ochrochloron* (ATCC 48663) were examined. The cultures were renewed monthly and stored at 4 °C. The microdilution method [44], was used to determine the minimum inhibitory concentration (MIC) and minimum fungicidal concentration (MFC) using a spore suspension concentration of (1.0 × 10^5^ CFU/mL) dilutions in 96-well microtiter plates. EOs were diluted to the desired concentrations in microplates containing Malt medium broth mixed with inoculum. The microplates were incubated at 28 °C for 72 h on a rotary shaker.

The lowest concentration that inhibits fungi growth at the binocular microscope level was defined as the MIC. The MFC was defined as the minimum concentration showing no visible growth, which is consistent with a 99.5% killing of the original inoculum. Serial sub-cultivations (2 μL) of essential oils were incubated at 28 °C for 72 h in microtiter plates containing 100 μL of broth and inoculum were used to calculate the MIC. Ketoconazole (KTZ) (1–3500 μg/mL) was used as a positive control. The experiments were performed in triplicate.

### Antioxidant activity of the EOs

To determine the free radical scavenging activity of the obtained EOs, the 2,2′-diphenypicrylhydrazyl (DPPH) method was employed [45] (absorbance at 517 nm), as along with the *β*-carotene-linoleic acid assay [8] (absorbance at 470 nm). A blank was prepared in the same manner as the samples and the antioxidant activities of the samples were compared with the blank and standard antioxidant, butylhydroxytoluene (BHT). All experiments were repeated twice in triplicates.

### Statistical analysis

The values of the antibacterial, antifungal, and antioxidant activities of EO from *C. citriodora* leaves and *C. macrocarpa* branchlets are presented as mean ± standard deviation (SD). Analysis of variance (ANOVA) was used to evaluate the differences between the groups. *P* < 0.05 was considered significant.

## Results

### Composition of the EOs

The EO of *Cupressus macrocarpa* branchlets contains 19 compounds (Table 1). The major chemical constituents identified included terpinen-4-ol (23.7%), *α*-phellandrene (19.2%), *α*-citronellol (17.3%), citronellal (6.6%), D-camphor (5.4%), *γ*-terpinene (5.3%), *α*-terpinene (3.08%), *α*-myrcene (2.2%), limonene (2.16%), *α*-terpineol (1.7%), terpinolene (1.8%), and *α*-linalool (1.5%).

**Table 1:** Essential oil constituents of *C. macrocarpa* branchlets and *C. citriodora* leaves

Constituent	RI^a,b^	Percentage *C. macrocarpa*	Percentage *C. citriodora*
α-Pinene	925	–	0.1
Thujene	953	0.9	0.4
*α*-Myrcene	985	2.2	0.1
*α*-Phellandrene	1005	19.2	–
*α*-Terpinene	1015	3.0	–
Eucalyptol/1,8-Cineole	1017	–	2.0
m-Cymene	1023	0.4	–
Limonene	1031	2.1	–
Terpinolene	1035	1.8	–
Melonal	1037	–	0.9
*γ*-Terpinene	1067	5.3	–
*α*-Linalool	1089	1.5	0.4
(+)-Rose oxide	1100	–	1.2
trans-2-menthenol	1140	1.0	–
Isopulegol	1145	–	7.6
D-Camphor	1151	5.4	–
Citronellal	1159	6.6	–
Borneol	1175	0.1	–
Terpinen-4-ol	1192	23.7	0.5
*α*-Terpineol	1193	1.7	0.2
Piperitol	1205	0.7	–
α-Citronellal	1211	–	56.0
*α*-Citronellol	1233	17.3	14.7
Citronellic acid	1305	1.3	1.4
Cubebol	1501	–	1.1
Citronellol acetate	1511	–	12.3
Spathulenol	1564	–	0.3
Caryophyllene oxide	1571	–	1.2
Farnesol	1696	0.4	–

^a^*RI* Retention Index

^b^Identification of the essential oil components was performed by comparison of mass spectra and RIs obtained in both columns with those of reference compounds and those of mass spectra libraries

Seventeen compounds were identified in the EO of *C. citriodora* leaves (Table 2). The major constituents were *α*-citronellal (56.0%), *α*-citronellol (14.7%), citronellol acetate (12.3%), isopulegol (7.6%), eucalyptol (2.0%), citronellic acid (1.4%), caryophyllene oxide (1.2%), and (+)-rose oxide (1.2%).

**Table 2:** Minimum inhibitory (MIC) and bactericidal concentration (MBC) of the essential oil (mg/mL) of *Corymbia citriodora* leaves Hook and *C. macrocarpa* branchlets

Bacterial strains	*Corymbia citriodora*		*Cupressus macrocarpa*		Streptomycin	
	MIC	MBC	MIC	MBC	MIC	MBC
*Agrobacterium tumefaciens*	0.16 ± 0.01d	0.37 ± 0.02b	0.23 ± 0.01b	0.55 ± 0.03b	0.23 ± 0.03a	0.43 ± 0.03a
*Bacillus cereus*	0.08 ± 0.01e	0.20 ± 0.01e	0.12 ± 0.01e	0.27 ± 0.03f	0.08 ± 0.02e	0.15 ± 0.01 g
*Dickeya solani*	0.18 ± 0.01b	0.29 ± 0.01d	0.21 ± 0.01c	0.38 ± 0.03c	0.09 ± 0.01e	0.19 ± 0.01d
*Escherichia coli*	0.06 ± 0.01f	0.12 ± 0.01f	0.07 ± 0.01f	0.15 ± 0.01 g	0.12 ± 0.01c	0.30 ± 0.03c
*Pectobacterium atrosepticum*	0.17 ± 0.01c	0.31 ± 0.01c	0.17 ± 0.02d	0.30 ± 0.03e	0.10 ± 0.01d,e	0.18 ± 0.00f
*Pectobacterium carotovorum*	0.16 ± 0.01d	0.29 ± 0.03c	0.13 ± 0.01e	0.31 ± 0.03d	0.12 ± 0.01c	0.22 ± 0.01e
*Staphylococcus aureus*	0.20 ± 0.01a	0.41 ± 0.03a	0.31 ± 0.01a	0.63 ± 0.05a	0.21 ± 0.01b	0.36 ± 0.03b

Means with the same letters within the same column are not significantly different (*p* < 0.05)

### Antibacterial activity

The MIC values of EO from *C. citriodora* leaves ranged from 0.06 mg/mL against *E. coli* to 0.20 mg/mL against *S. aureus*, and those values were lower than the MIC values of streptomycin (Table 2). Additionally, activity of this oil was comparable or even higher than reference antibiotic in case of *Agrobacterium tumefaciens* or *B. cereus*. EO from *C. macrocarpa* branchlets showed less activity against bacterial strains. The MIC values ranged from 0.07 mg/mL against *E. coli* to 0.31 mg/mL against *S. aureus*. The MBC values of EO from *C. citriodora* ranged from 0.12 mg/mL against *E. coli* to 0.41 mg/mL against *S. aureus*, whereas, the values were between 0.15 mg/mL (*E. coli*) and 0.63 mg/mL (*S. aureus*) using EO from *C. macrocarpa* branchlets. The EO of *C. citriodora* and *C. macrocarpa* showed noticeable activity against phytopathogenic bacteria including *Pectobacterium atrosepticum*, *P. carotovorum,* and *Dickeya solani*, which causes many diseases in potato production, such as the blackleg in the field and soft rot during storage. Furthermore, all MIC values reported against the potato pathogenic bacteria were lower than those reported for the negative control, streptomycin.

### Antifungal activity

The antifungal activities of the EOs against several fungi are shown in Table 3. The MIC values of the EO from *C. citriodora* ranged from 0.11 mg/mL (*A. niger*) to 0.52 mg/mL (*P. funiculosum*), while the MFC values ranged from 0.25 mg/mL (*A. niger*) to 0.95 mg/mL (*P. funiculosum*). The MIC values of the EO from *C. macrocarpa* ranging from 0.29 mg/mL (*P. ochrochloron*) to 3.21 mg/mL (*C. albicans*), and the MFC values ranged from 0.53 mg/mL (*P. ochrochloron*) to > 5 mg/mL (*C. albicans*). It was noted that the MIC and MFC values of EOs against *P. funiculosum* were lower than those obtained from KTZ. In addition, the EO from *C. citriodora* leaves showed more potency than the EO of *C. macrocarpa* needles against the tested fungi.

**Table 3:** Minimum inhibitory concentration (MIC) and fungicidal concentration (MFC) of different leaf extracts (mg/mL) of *Corymbia citriodora* and *Cupressus macrocarpa*

Fungal strains	*Corymbia citriodora*		*Cupressus macrocarpa*		KTZ	
	MIC	MFC	MIC	MFC	MIC	MFC
*Aspergillus flavus*	0.21 ± 0.01f	0.46 ± 0.03f	0.31 ± 0.03 h	0.75 ± 0.03d	0.23 ± 0.01c	0.48 ± 0.05c
*Aspergillus ochraceus*	0.26 ± 0.03d	0.51 ± 0.01e	0.54 ± 0.03f	1.43 ± 0.10c	0.22 ± 0.01d	0.43 ± 0.03d
*Aspergillus niger*	0.11 ± 0.03 h	0.25 ± 0.03 h	1.25 ± 0.13c	> 2b	0.10 ± 0.03 g	0.22 ± 0.00 g
*Candida albicans*	0.25 ± 0.01e	0.52 ± 0.01d	3.21 ± 0.15a	> 5a	0.21 ± 0.01e	0.43 ± 0.03d
*Fusarium oxysporum*	0.27 ± 0.01c	0.58 ± 0.03c	1.11 ± 0.05d	> 2b	0.26 ± 0.01b	0.52 ± 0.03b
*Penicillium funiculosum*	0.52 ± 0.05a	0.95 ± 0.05a	0.73 ± 0.05e	1.47 ± 0.07c	2.01 ± 0.11a	3.65 ± 0.01a
*Penicillium ochrochloron*	0.20 ± 0.01 g	0.45 ± 0.03 g	0.29 ± 0.01 g	0.53 ± 0.03e	0.21 ± 0.01e	0.41 ± 0.03f
*Rhizoctonia solani*	0.37 ± 0.01b	0.81 ± 0.05b	> 2b	N.D.	0.19 ± 0.01f	0.42 ± 0.03e

*N.D.* Not detected

*KTZ* Ketoconazole

Means with the same letters within the same column are not significantly different (*p* < 0.05)

### Antioxidant activity

The antioxidant activity of tested EOs (IC_50_ = 5.1 ± 0.1 and 6.1 ± 0.1 μg/mL for *C. citriodora* and *C. macrocarpa*, respectively) is lower than that of the standard BHT (IC_50_ = 2.9 ± 0.2 μg/mL) (Table 4).

**Table 4:** Essential oils DPPH and *β*-carotene-linoleic acid assay results

Sample	IC50, μg/mL	
	DPPH assay	β-Carotene-linoleic acid assay
*Corymbia citriodora*	5.1 ± 0.1b	3.2 ± 0.1b
*Cupressus macrocarpa*	6.1 ± 0.1c	4.0 ± 0.1c
BHT	2.9 ± 0.2a	2.6 ± 0.1a

Values are expressed as mean of triplicate determinations ± standard deviation

*BHT* Butylated hydroxyltoluene

Means with the same letters within the same column are not significantly different (*p* < 0.05)

## Discussion

Giatropoulos et al. [46] reported that sabinene (21.8%), *α*-pinene (19.5%), terpinen-4-ol (18.9%), *γ*-terpinene (7.9%), and *α*-terpinene (5.7%) were the major components detected in the needle EO of *C. macrocarpa* grown in Athens, Greece. In India, the major compounds identified in EO of cones of *C. macrocarpa* were terpinel-4-ol (19.4%), dinopol (15.6%), *α*-pinene (13.5%), and *β*-pinene (12.1%) [11]. Recent investigation of Fahed et al. [19] found similar major essential oil constitutes such as sabinene and terpinen-4-ol. The primary compounds in the volatile oils of fresh and dried leaves of *C. macrocarpa* (gold crest) grown at the north coast of Egypt were neral (31–35%), hydroxy citronellal (12–16%), geraniol (3–4%), piperitol (*trans*) (7–8%), isobornyl isobutyrate (0.7–6.6%), linalool (0.6–5.2%), terpinyl acetate (0.10–3.2%), myrcene (0.22–2.6%), *trans*-ferruginol (0.3–2.25%), abitol (0.4–2.18%), and eugenol dihydro (0.1–1.3%) [17]. In Argentina, *C. macrocarpa* oil was found to be composed of *α*-pinene (20.2%), sabinene (12.0%), *p*-cymene (7.0%), and terpinen-4-ol (29.6%) [15]. *C. macrocarpa* oils are rich in sabinene (27.0, 23.3%), *α*-pinene (22.2, 19.8%), and terpinen-4-ol (11.7, 14.7%) with moderate amounts of *γ*-terpinene (5.6, 5.1%), isophyllocladene (4.4, 4.9%), myrcene (3.6, 3.2%), *β*-pinene (2.6, 2.0%), and phyllocladene (2.3, 2.0%) [47].

In agreement with our results, Jang et al. [48] found that the major EOs constitutes of *C. citriodora* are α-citronellal and isopulegol. Singh et al. [26] found that the major monoterpenoids detected in the EO of *C. citriodora* were citronellal (60.6%), *β*-citronellol (12.5%), and isopulegol (8.1%). In addition, citronellal and *β*-citronellol were the major components in the leaf EO of *C. citriodora* [28]. However, the major component of the leaves of *C. citriodora* grown at the State of Ceará, Brazil, was *β*-citronellal (71.7%) [49]. In contrast, 6-octenal (77.1%) was found to be a major component in the EO of *C. citriodora* grown in Nigeria [26], and *α*-pinene (38.6%), *β*-pinene (25.6%), sabinene (19.6%), and *α*-thujene (11.9%) were the major compounds contained in the EO of *C. citriodora* leaves from Paschim Vihar (New Delhi) [29]. *Neo*-isopulegol, citronellal, *iso*-isopulegol, citronellol, citronellyl acetate, and *E*-caryophyllene were the primary components in the EO of the plant from Benin [50]. Hussein et al. [51] found that α-citronellal, α-citronellol, citronellol acetate, and isopulegol were the major chemical constituents from *C. citriodora* leaf EO. 1.8-cineole and *α*-pinene were the primary components in the EO from *C. citriodora* grown in Zerniza and Souinet Arboreta (North West and North Tunisia) [52]. Interestingly, 6-octenal was not found in our study.

Citronellal and citronellol found in the EO of *C. citriodora* may be responsible for both its antimicrobial activity and antioxidant activity [24, 26, 53, 54]. Elaissi et al. [52] reported inhibition zone values ranging from 10.0 ± 0.0 mm to 7.7 ± 0.6 mm against *E. coli* ATCC 25922 and *S. aureus* ATCC 25932, respectively, using absorbent disks impregnated with 10 μL of *C. citriodora* oil.

The EO from *C. citriodora* showed higher antifungal activity than the positive control. These results are consistent with those of Ramezani et al. [22], who found that the volatile oil is more potent than the synthetic fungicide Mancozeb, and that *C. citriodora* oil strongly inhibits radial growth of *Macrophomina phaseolina*, *Colletotrichum lindemuthianum*, *Fusarium oxysporum* f. *sp. lycopersici*, *Helminthosporium oryzae*, *Alternaria triticina*, *Rhizoctonia solani,* and *Alternaria solani* with MICs ranging between 0.25 and 0.50 ppm. Fahed et al. [19] reported strong antifungal activities of the EOs of *C. macrocarpa* against specific fungi such as *Trichophyton rubrum* and it was associated mainly with major essential oil constitutes such as sabinene and terpinen-4-ol.

We found that The antioxidant activity are differed from those previously reported using the hydro-distillated EOs from the Indian *C. citriodora* with an IC_50_ of 425.4 ± 6.79 μg/mL (DPPH) and 87.3 ± 9.27 μg/mL (reduced activity) [26]. EO from *C. citriodora* leaves is rich in monoterpenoids and thus, shows strong antioxidant activity [26, 53, 54]. 

It was concluded that the volatile oils of *C. citriodora* may have tremendous potential as antimicrobial agents in food sciences in addition to their numerous uses and applications in pharmaceutical and medicinal areas [55].

## Conclusions

The EO of *C. macrocarpa* branchlets primarily comprised terpinen-4-ol, *α*-phellandrene, *α*-citronellol, and citronellal, while in *C. citriodora* the oil consisted primarily of *α*-citronellal, *α*-citronellol, citronellol acetate, isopulegol, and eucalyptol. Moderate activity was found against the studied bacterial strain. However, the EO of *C. citriodora* leaves showed more potency than the *C. macrocarpa* branchlets did against the studied fungi. The EO of *C. citriodora* showed higher activity than the positive control did. Additionally, the antioxidant activity of tested EOs was lower than that of the standard BHT used.

## Abbreviations

ANOVA: Analysis of variance
ATCC: American Type Culture Collection
BHT: Butylhydroxytoluene
CFU: Colony-forming unit
DMSO: Dimethyl sulfoxide
DPPH: 2,2′-diphenypicrylhydrazyl
EI: Electron impact ionization
EO: Essential oil
FID: Flame Ionization Detector
GC/MS: Gas chromatography–mass spectrometry
HP: Hewlett Packard
IC_50_: The concentration required to inhibit DPPH radical formation by 50%
KTZ: Ketoconazole
MBC: Minimum bactericidal concentration
MFC: Minimum fungicidal concentration
MIC: Minimum inhibitory concentration
RI: Retention index
SD: Standard deviation
TSB: Triptic Soy broth
